# Long-term suicide risk in lung cancer patients: a nationwide cohort study

**DOI:** 10.1017/S2045796026100870

**Published:** 2026-08-11

**Authors:** Jung Eun Yoo, Jin-Hyung Jung, Kyung-Do Han, Dong Woog Yoon, Seonghye Kim, Ji Hyun An, Jong Ho Cho, Dong Wook Shin

**Affiliations:** 1 Department of Family Medicine, Healthcare System Gangnam Center, Seoul National University Hospital, Seoul, Republic of Korea; 2 Department of Family Medicine, Seoul National University College of Medicine, Seoul, Republic of Korea; 3 Health Insurance Review and Assessment Service, Wonju-si, Gangwon-do, Republic of Korea; 4 Department of Statistics and Actuarial Science, Soongsil University, Seoul, Republic of Korea; 5 Department of Thoracic and Cardiovascular Surgery, Chung-Ang University Hospital, Seoul, Republic of Korea; 6 Supportive Care Center, Department of Family Medicine, Samsung Medical Center, Sungkyunkwan University School of Medicine, Seoul, Republic of Korea; 7 Department of Psychiatry, Depression Center, Samsung Medical Center, Sungkyunkwan University School of Medicine, Seoul, Republic of Korea; 8 Department of Thoracic and Cardiovascular Surgery, Samsung Medical Center, Sungkyunkwan University School of Medicine, Seoul, Korea; 9 Department of Clinical Research Design & Evaluation, Samsung Advanced Institute for Health Science & Technology (SAIHST), Sungkyunkwan University, Seoul, Republic of Korea

**Keywords:** adjuvant therapy, landmark analysis, lung cancer, suicide, surgery

## Abstract

**Aims:**

Lung cancer patients have a higher risk of suicide, but the long-term patterns and how they differ by treatment type have not been fully characterized. To investigate longitudinal suicide risk among lung cancer patients who underwent surgery compared to matched controls and to examine variations over time by treatment modality and patient characteristics.

**Methods:**

We identified 37,215 individuals newly diagnosed with lung cancer between 2010 and 2017 who underwent lung resection within 1 year of diagnosis, along with 1:3 age- and sex-matched controls. Subdistribution hazard ratios (sHRs) and 95% confidence intervals (CIs) for suicide were estimated using a competing risk analysis.

**Results:**

The cohort (mean age 64.0 years; 62.5% male) was followed for a mean of 7.6 years. There were 154 suicides among lung cancer patients (incidence rate 0.6 per 1,000 person-years). Suicide risk peaked during the first year after surgery (sHR 1.88, 95% CI 1.23–2.87) and declined in subsequent years. Patients who underwent surgery alone had a higher risk in the first year (sHR 1.66, 95% CI 1.02–2.69), which gradually decreased over time. In contrast, patients treated with adjuvant chemotherapy or radiotherapy experienced a substantially higher initial risk (e.g., sHR 7.00, 95% CI 3.00–16.37 for surgery + radiotherapy) and maintained a moderate increase in suicide risk even beyond 5 years. Subgroup analyses showed an especially elevated first-year suicide risk among younger adults aged 20–49 years, males, and never smokers.

**Conclusions:**

Suicide risk for lung cancer patients is highest during the first postoperative year, particularly for those who receive multimodal therapy, or are younger adults, males, or never smokers. These findings emphasize the need to prioritize early, tailored psychosocial support in survivorship care to help mitigate long-term suicide risk.

## Introduction

A diagnosis of cancer is commonly linked to a markedly increased risk of suicide (Zaorsky *et al.*, 2019). This increased vulnerability results from a multifactorial interplay that includes the existential threat posed by the diagnosis, severe physical symptoms, treatment-associated adverse effects, and significant psychosocial distress. Among various cancer types, lung cancer consistently exhibits one of the highest risks of suicide (Zaorsky *et al.*, 2019). Standardized mortality ratios (SMRs) for suicide among lung cancer patients have been reported within a range of approximately 3.0–4.2, with the greatest risk noted in the first year following diagnosis (Rahouma *et al.*, 2018; Zaorsky *et al.*, 2019; Tang *et al.*, 2022; Hofmann *et al.*, 2023). In one study, SMRs as high as 25 were observed during this initial period (Zaorsky *et al.*, 2019). Despite growing awareness of this short-term increase, there remain few comprehensive data describing the longitudinal pattern of suicide risk in lung cancer. A recent scoping review indicated that excess suicide mortality peaks within the first year after diagnosis (SMR 2.04–13.4) and tends to decrease in subsequent years (SMR 0.66–3.17) (Tang *et al.*, 2022). Yet, most studies to date have relied on SMRs derived from death registry data with limited adjustment for behavioural or socioeconomic variables (Tang *et al.*, 2022). Because these SMR-based comparisons do not adequately control for unmeasured differences between cancer cohorts and reference populations, there is a clear need for prospective, covariate-adjusted analyses in rigorously defined cohorts.

Previous research has primarily addressed general comparisons between surgical and non-surgical strategies or considered the psychological impact of chemotherapy and radiotherapy in cancer patients (McFarland *et al.*, 2019; Potter *et al.*, 2023). Nevertheless, limited studies have provided detailed analyses of suicide risk among lung cancer patients by specific treatment modalities over time. For example, one population-based investigation found no long-term difference in suicide incidence between surgical and non-surgical lung cancer patients, but its conclusions were limited by the small sample size (1,306 surgical and 3,189 non-surgical lung cancer patients) and the absence of treatment classification within the non-surgical group (Kim *et al.*, 2025). Moreover, understanding the influence of demographic variables such as age and sex on longitudinal suicide risk is vital for developing tailored survivorship care and psychosocial strategies.

Based on these considerations, we sought to investigate suicide risk among lung cancer patients in comparison to age- and sex-matched controls, while controlling for relevant confounders. Furthermore, we characterized the temporal evolution of suicide risk and analysed differences in risk according to treatment type and individual patient attributes.

## Methods

### Data source and study setting

The National Health Insurance Service (NHIS) in Korea is a mandatory, universal health insurance system that covers approximately 97% of the population, whereas the remaining 3%, primarily low-income groups, are supported through the government-funded Medical Aid program. The NHIS database includes extensive information on demographic variables, medical interventions, prescribed medications, and disease diagnoses based on the International Classification of Diseases, 10th Revision (ICD-10) codes. In addition, the NHIS provides a biennial national health screening program for all eligible Korean citizens, incorporating self-administered questionnaires covering medical history, health-related behaviours (such as smoking, alcohol intake, and physical activity), as well as anthropometric parameters (e.g., body mass index) and laboratory test results. (Shin *et al.*, 2022)

### Study population

**Figure 1.**
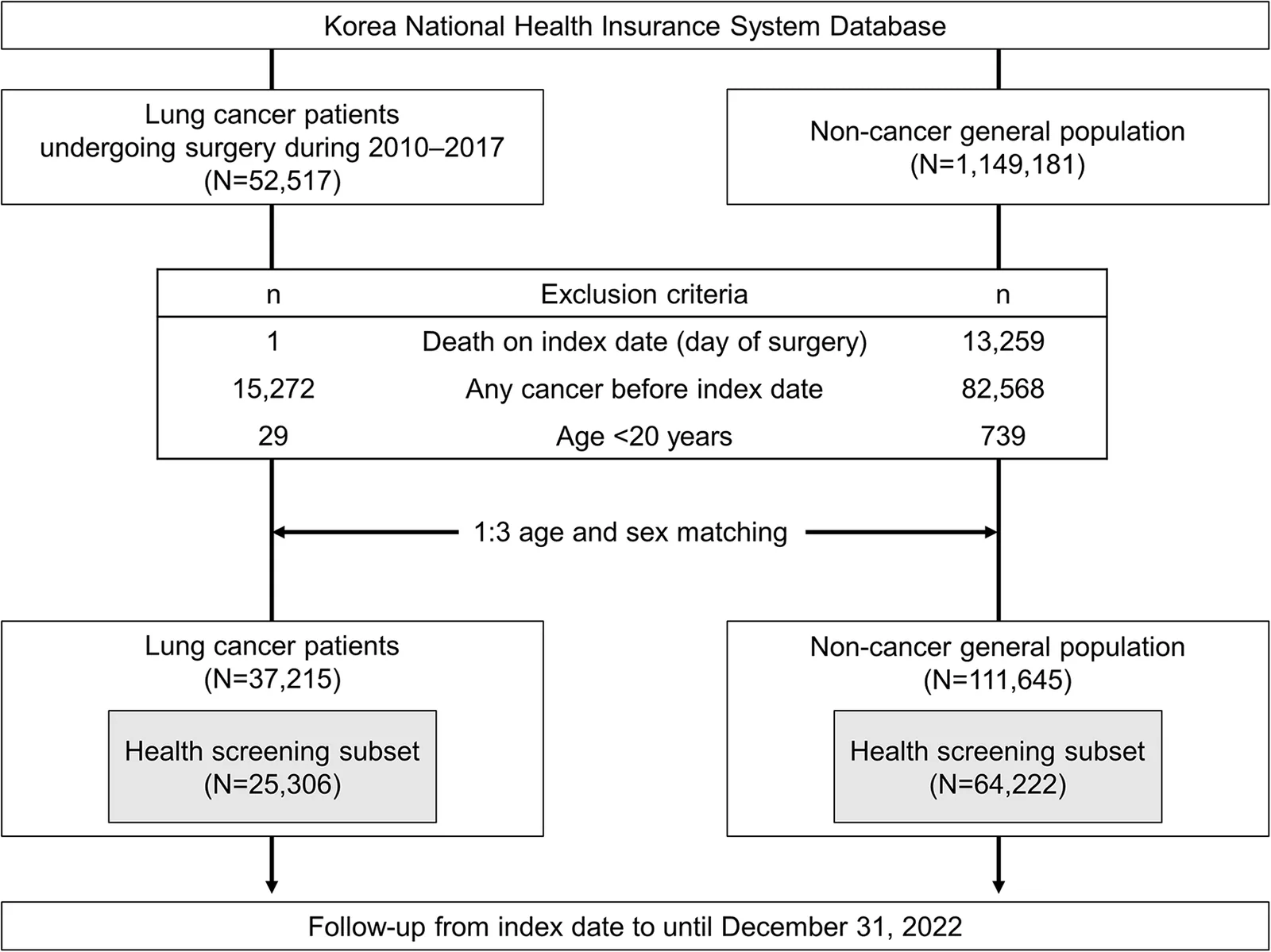
Study population flow chart.

We initially identified 52,517 patients newly diagnosed with lung cancer (ICD-10 codes C33–34) between 2010 and 2017 who underwent lung resection within 1 year of diagnosis. To ensure diagnostic accuracy, only patients enrolled in the critical illness co-payment reduction program (V193), which mandates a physician-certified diagnosis and reduces out-of-pocket cancer-related medical costs to 5%, were included, following the methodology of previous studies (Lee *et al.*, 2019; Jeon *et al.*, 2020). We excluded patients with lung cancer who did not undergo lung resection surgery to eliminate late-stage or inoperable cases from the analysis. Patients were also excluded if they (1) died on the index date (day of surgery) (*n* = 1), (2) had a prior cancer diagnosis (*n* = 15,272), or (3) were younger than 20 years of age (*n* = 29). The final lung cancer cohort comprised 34,519 patients (Fig. 1). The control cohort (*n* = 111,645) was established through 1:3 matching based on age and sex among individuals without a cancer diagnosis.

A health screening subset was formed from participants who had undergone health screening within 2 years preceding their index date, totalling 25,306 lung cancer patients and 64,222 controls, for whom data on anthropometric characteristics and health behaviours were available.

This study received approval from the Institutional Review Board of Samsung Medical Centre (IRB No. SMC 2024-05-113), and the requirement for informed consent was waived due to the exclusive use of de-identified administrative data.

### Outcomes and follow-up

The primary outcome of this study was suicide, identified using ICD-10 codes X60–X84 from the national death registration database managed by the Korean National Statistical Office. This registry records the date, cause, and place of death and is based on death certificates certified by physicians in over 90% of cases (Shin *et al.*, 2010). Follow-up began at the index date and continued until the occurrence of suicide or the last follow-up (31 December 2022), whichever came first.

### Covariates

Potential confounders were comprised of demographic variables, including age, sex, income level, and residential area, as well as comorbidities such as hypertension (ICD-10 codes I10–I13 or I15 with antihypertensive medication), diabetes mellitus (E11–E14 with antidiabetic medication), dyslipidemia (E78 with lipid-lowering agent), ischemic heart disease (I20–I25), stroke (I63–I64), chronic obstructive pulmonary disease (J41–J44), end-stage kidney disease (identified by registration in the critical illness co-payment reduction program for hemodialysis [V001], peritoneal dialysis [V003], or kidney transplant [V005]), and depression (F32–F33).

Within the health screening subset, further covariates incorporated body mass index (BMI), which was calculated as weight divided by height squared (kg/m^2^); smoking status, defined as never, former, or current smoker and further categorized based on <20 or ≥20 pack-years; alcohol consumption, determined by any reported alcohol intake; and regular physical activity, assessed as more than 30 minutes of moderate-intensity exercise at least five times per week or more than 20 minutes of vigorous-intensity exercise at least three times per week.

### Statistical analysis

Baseline characteristics are summarized as the mean ± standard deviation for continuous variables or as number (%) for categorical variables. Suicide incidence rates (IRs) were determined per 1,000 person-years. To account for the competing risk of non-suicide death, the Fine-Gray subdistribution hazard model was used to obtain subdistribution hazard ratios (sHRs) with 95% confidence intervals (CIs). Models were adjusted for the following confounders: age, sex, income, place of residence, hypertension, diabetes mellitus, dyslipidemia, ischemic heart disease, stroke, chronic obstructive pulmonary disease, end-stage kidney disease, and depression. In the health screening subsets, analyses were further adjusted for BMI, smoking, alcohol consumption, and regular physical activity. Lung cancer patients were stratified into four treatment groups: (1) surgery only, (2) surgery + chemotherapy, (3) surgery + radiotherapy, and (4) surgery + chemotherapy + radiotherapy. To assess temporal changes in suicide risk, landmark analyses were performed for the first year following surgery, as well as with lag times of 1, 3, and 5 years, facilitating comparison of short- and long-term risk trajectories. Subgroup analyses, conducted according to age, sex, and smoking status, incorporated interaction terms tested using likelihood-ratio tests.

All statistical analyses were performed with SAS version 9.4 (SAS Institute Inc., Cary, NC, USA). A two-sided *P-*value < 0.05 was considered statistically significant.

## Results

### Baseline characteristics

Table 1 summarizes the baseline characteristics of the study participants. The mean age of the participants was 64.0 ± 10.0 years, and 65.2% were male. Compared to their matched controls, lung cancer patients were more frequently from the highest income quartile and predominantly lived in urban areas. In addition, these patients had a greater prevalence of comorbid conditions, such as hypertension, diabetes mellitus, dyslipidemia, ischemic heart disease, stroke, chronic obstructive pulmonary disease, and depression. In addition, lung cancer patients in the health screening subset showed a higher prevalence of current heavy smoking and comorbidities compared to matched controls (Table S1).

**Table 1.** Baseline characteristics of the study population Table 1 long description.

Variables	Lung cancer patients (*N* = 37,215)	Matched controls (*N* = 111,645)	*P* value
Age (years)	64.0 ± 10.0	64.0 ± 10.0	-
20−49	11,716 (7.9)	2,929 (7.9)	-
50−69	88,472 (59.4)	22,118 (59.4)	
≥70	48,672 (32.7)	12,168 (32.7)	
Sex			
Male	23,261 (62.5)	69,783 (62.5)	-
Female	13,954 (37.5)	41,862 (37.5)	
Income (quartiles)			
Q1 (lowest 25% + medical aid)	7,498 (20.2)	25,318 (22.7)	< 0.001
Q2	5,813 (15.6)	19,212 (17.2)	
Q3	8,521 (22.9)	26,172 (23.4)	
Q4	15,383 (41.3)	40,943 (36.7)	
Place of residence			
Rural	20,219 (54.3)	61,473 (55.1)	0.014
Urban	16,996 (45.7)	50,172 (44.9)	
Comorbidities			
Hypertension	17,296 (46.5)	46,187 (41.4)	<0.001
Diabetes mellitus	6,870 (18.5)	17,385 (15.6)	<0.001
Dyslipidemia	11,989 (32.2)	27,851 (25.0)	<0.001
Ischemic heart disease	7,062 (19.0)	12,322 (11.0)	<0.001
Stroke	2,464 (6.6)	59,41 (5.3)	<0.001
Chronic obstructive pulmonary disease	15,634 (42.0)	10,914 (9.8)	<0.001
End-stage kidney disease	171 (0.5)	429 (0.4)	0.047
Depression	3,969 (10.7)	8,557 (7.7)	<0.001
Lung cancer treatment			
Surgery only	23,982 (64.4)	-	-
Surgery + CTx	8,197 (22.0)	-	-
Surgery + RTx	1,157 (3.1)	-	-
Surgery + CTx + RTx	3,879 (10.4)	-	-

CTx, chemotherapy; RTx, radiotherapy.

Data are expressed as the means ± standard deviation or number (%).

### Time-varying suicide risk in lung cancer patients

**Figure 2.**
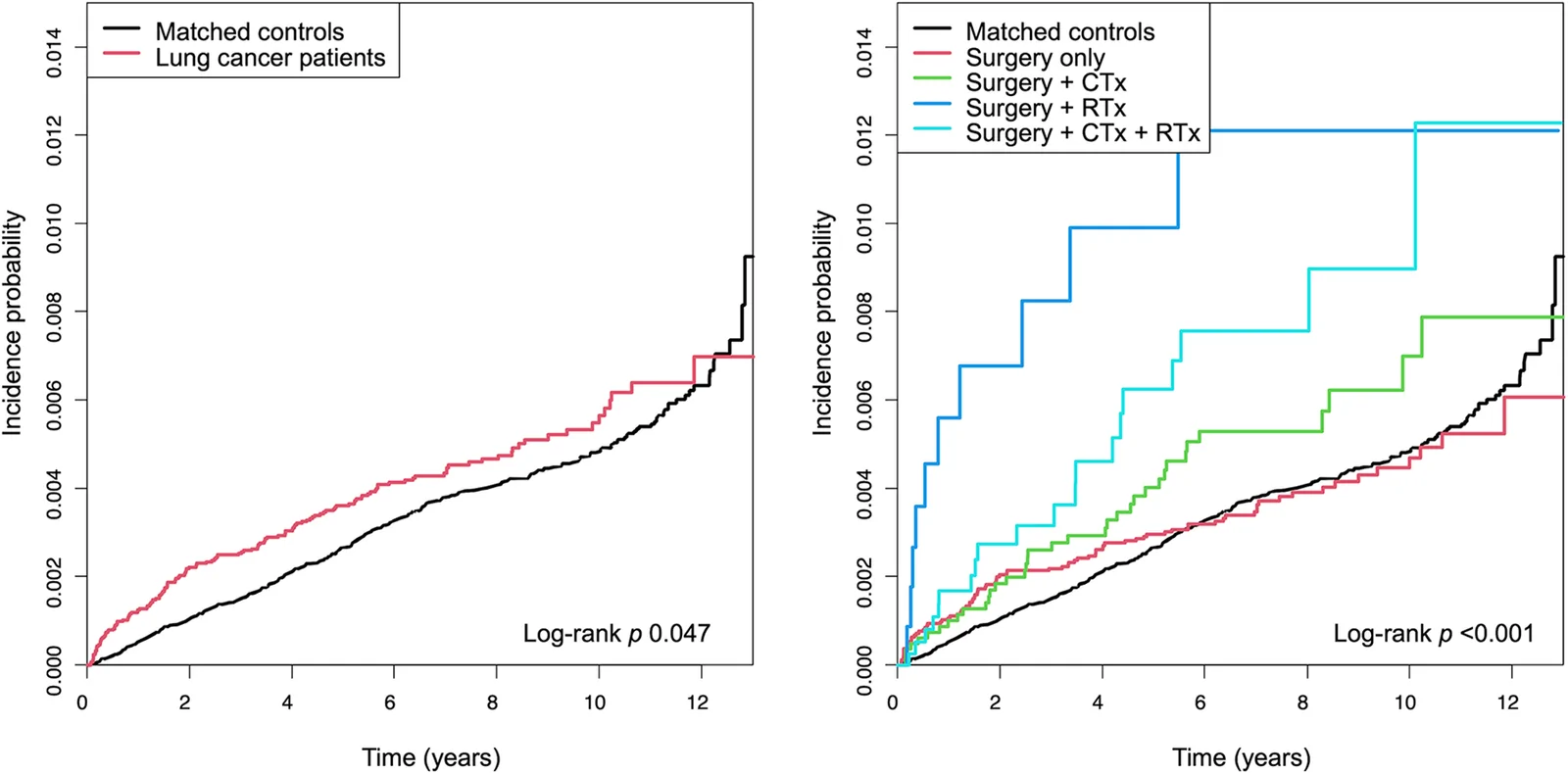
Cumulative incidence curves depicting suicide rates among lung cancer patients. CTx, chemotherapy; RTx, radiotherapy.

**Figure 3.**
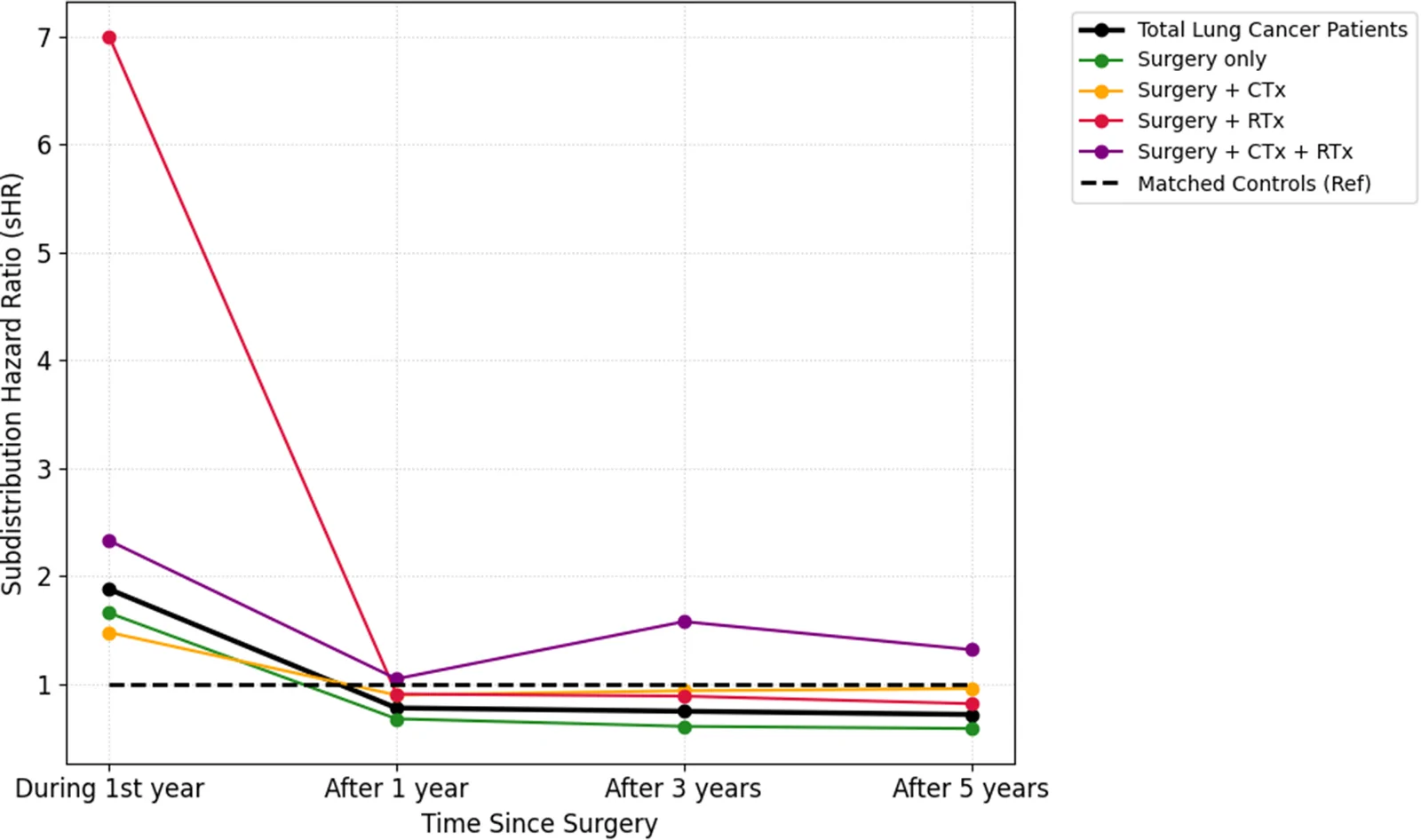
Time-varying suicide risk trajectories by treatment modality in lung cancer patients following surgery. CTx, chemotherapy; RTx, radiotherapy.

Over a mean follow-up period of 7.6 ± 2.9 years, 154 suicides occurred among lung cancer patients (IR 0.6 per 1,000 person-years), compared with 468 suicides in the matched controls (IR 0.5 per 1,000 person-years; Table 2). Cumulative incidence function (CIF) curves demonstrated a higher cumulative incidence of suicide in lung cancer patients than in controls (Fig. 2). The difference in risk became apparent early, with the most marked increase seen within the first year after surgery (Fig. 3). Landmark analysis indicated a markedly increased suicide risk during this timeframe (sHR 1.88, 95% CI 1.23–2.87). Afterwards, the risk declined, with corresponding sHRs (95% CI) of 0.78 (0.62–0.97), 0.75 (0.57–1.00), and 0.72 (0.50–1.03) at 1, 3, and 5 years, respectively. Comparable temporal patterns were also observed in the health screening subset.

**Table 2.** Risk of suicide in lung cancer patients according to year since lung cancer surgery Table 2 long description.

	Overall			During 1^st^ year	After 1 year	After 3 years	After 5 years
	N	Event/IR	sHR* (95% CI)	sHR* (95% CI)	sHR* (95% CI)	sHR* (95% CI)	sHR* (95% CI)
Total population							
Matched controls	111,645	468/0.5	1 (ref.)	1 (ref.)	1 (ref.)	1 (ref.)	1 (ref.)
Lung cancer patients	37,215	154/0.6	0.87 (0.71−1.06)	1.88 (1.23−2.87)	0.78 (0.62−0.97)	0.75 (0.57−1.00)	0.72 (0.50−1.03)
Surgery only	23,982	87/0.5	0.78 (0.61−0.99)	1.66 (1.02−2.69)	0.68 (0.52−0.91)	0.61 (0.43−0.86)	0.59 (0.37−0.92)
Surgery + CTx	8,197	37/0.8	0.92 (0.65−1.29)	1.48 (0.68−3.23)	0.90 (0.61−1.33)	0.94 (0.58−1.53)	0.96 (0.51−1.82)
Surgery + RTx	1,157	10/1.9	1.51 (0.80−2.83)	7.00 (3.00−16.37)	0.91 (0.34−2.45)	0.89 (0.22−3.60)	0.82 (0.11−6.06)
Surgery + CTx + RTx	3,879	20/1.2	1.05 (0.66−1.67)	2.33 (0.94−5.79)	1.05 (0.61−1.81)	1.58 (0.83−3.01)	1.32 (0.48−3.59)
Health screening subset							
Matched controls	64,222	237/0.5	1 (ref.)	1 (ref.)	1 (ref.)	1 (ref.)	1 (ref.)
Lung cancer patients	25,306	91/0.6	0.8 (0.61−1.04)	1.91 (1.07−3.41)	0.71 (0.52−0.96)	0.70 (0.49−1.01)	0.67 (0.43−1.05)
Surgery only	16,300	55/0.5	0.76 (0.56−1.04)	1.87 (0.98−3.54)	0.65 (0.45−0.93)	0.55 (0.35−0.87)	0.52 (0.29−0.92)
Surgery + CTx	5,606	21/0.6	0.82 (0.52−1.30)	1.46 (0.48−4.46)	0.80 (0.49−1.33)	0.90 (0.50−1.61)	1.06 (0.52−2.16)
Surgery + RTx	707	3/0.9	0.70 (0.22−2.22)	4.09 (1.01−16.64)	0.34 (0.05−2.50)	0.67 (0.09−4.93)	1.16 (0.15−8.90)
Surgery + CTx + RTx	2,693	12/1.0	0.98 (0.53−1.80)	2.37 (0.66−8.55)	0.99 (0.49−1.99)	1.56 (0.71−3.44)	0.89 (0.22−3.67)

IR, incidence rate per 1,000 person-years; sHR, subdistribution hazard ratio; CI, confidence interval; CTx, chemotherapy; RTx, radiotherapy.

* Adjusted for age, sex, income, place of residence, hypertension, diabetes mellitus, dyslipidemia, ischemic heart disease, stroke, chronic obstructive pulmonary disease, end-stage kidney disease, and depression. For health screening subset, BMI, smoking, alcohol consumption, and regular physical activity were additionally adjusted.

### Suicide risk by treatment modality

When analysed by treatment modality, patients in the surgery-only group experienced an elevated suicide risk during the first post-surgical year (sHR 1.66, 95% CI 1.02–2.69), with risk declining progressively over time and falling below that of controls beyond 5 years (sHR 0.59, 95% CI 0.37–0.92). Individuals treated with adjuvant chemotherapy or radiotherapy showed a more pronounced early increase in suicide risk. For example, lung cancer patients with surgery + radiotherapy in the health screening subset had an elevated suicide risk (sHR 4.09, 95% CI 1.01–16.64) during the first post-surgical year, and although risk estimates for these groups decreased over time, they remained elevated beyond 5 years (sHR 1.16, 95% CI 0.15–8.90). However, these long-term estimates should be interpreted with caution, as wide CIs preclude definitive conclusions. Treatment-specific CIF curves revealed similar risk trends.

### Subgroup analysis

In the age-stratified analysis, patients aged 20–49 years demonstrated the highest baseline suicide risk (sHR 2.02, 95% CI 0.92–4.41, *P* for interaction 0.018), which remained relatively increased throughout the follow-up period (e.g., sHR 1.90, 95% CI 0.77–4.65 after 1 year, *P* for interaction 0.020; sHR 1.61, 95% CI 0.30–8.80 after 5 years) (Table 3, Figure S1). Both male and female patients had increased suicide risk in the initial year following surgery, with the association being more pronounced among males (sHR 1.93, 95% CI 1.22–3.06). Within the health screening subset, never-smokers exhibited higher suicide risk during the first postoperative year (sHR 2.21, 95% CI 1.01–4.87), with a generally decreasing trend observed in all smoking status groups as time progressed.

**Table 3.** Risk of suicide in lung cancer patients according to age, sex, and smoking Table 3 long description.

		Overall	During 1^st^ year	After 1 year	After 3 years	After 5 years
		sHR* (95% CI)	sHR* (95% CI)	sHR* (95% CI)	sHR* (95% CI)	sHR* (95% CI)
Total population						
Age	20−49	2.02 (0.92−4.41)	2.62 (0.52−13.13)	1.90 (0.77−4.65)	1.18 (0.31−4.44)	1.61 (0.30−8.80)
	50−69	0.97 (0.75−1.27)	1.68 (0.93−3.02)	0.90 (0.67−1.22)	0.90 (0.63−1.29)	0.88 (0.55−1.41)
	≥70	0.68 (0.51−0.91)	2.04 (1.14−3.66)	0.57 (0.40−0.81)	0.56 (0.36−0.89)	0.48 (0.26−0.89)
	*P* for interaction	0.018	0.813	0.020	0.213	0.201
Sex	Male	0.86 (0.69−1.06)	1.93 (1.22−3.06)	0.77 (0.60−0.99)	0.79 (0.58−1.07)	0.73 (0.49−1.08)
	Female	0.91 (0.58−1.44)	1.65 (0.63−4.31)	0.80 (0.47−1.35)	0.57 (0.27−1.22)	0.67 (0.28−1.61)
	*P* for interaction	0.807	0.769	0.916	0.431	0.872
Health screening subset						
Age	20−49	1.60 (0.36−7.20)	2.20 (0.14−35.29)	1.43 (0.24−8.61)	0.78 (0.08−7.55)	2.39 (0.15−38.81)
	50−69	0.99 (0.70−1.39)	2.27 (1.03−5.05)	0.87 (0.60−1.28)	0.84 (0.54−1.30)	0.77 (0.44−1.37)
	≥70	0.57 (0.38−0.85)	1.54 (0.69−3.45)	0.50 (0.31−0.80)	0.52 (0.29−0.94)	0.48 (0.22−1.03)
	*P* for interaction	0.067	0.778	0.133	0.438	0.416
Sex	Male	0.80 (0.59−1.07)	1.91 (0.99−3.69)	0.72 (0.52−1.01)	0.74 (0.50−1.10)	0.69 (0.42−1.15)
	Female	0.80 (0.45−1.42)	1.91 (0.59−6.26)	0.64 (0.33−1.26)	0.53 (0.22−1.29)	0.60 (0.22−1.61)
	*P* for interaction	0.999	0.997	0.743	0.494	0.798
Smoking	Never	0.82 (0.55−1.24)	2.21 (1.01−4.87)	0.65 (0.40−1.06)	0.55 (0.29−1.04)	0.44 (0.19−1.04)
	Former	0.95 (0.60−1.52)	2.48 (0.89−6.86)	0.83 (0.49−1.43)	0.84 (0.43−1.63)	0.71 (0.29−1.73)
	Current < 20PY	0.77 (0.36−1.67)	1.60 (0.33−7.71)	0.69 (0.28−1.71)	0.68 (0.23−1.99)	0.73 (0.16−3.34)
	Current ≥ 20PY	0.63 (0.38−1.07)	0.90 (0.20−4.07)	0.68 (0.39−1.19)	0.80 (0.43−1.51)	0.96 (0.43−2.11)
	*P* for interaction	0.711	0.708	0.916	0.784	0.622

sHR, subdistribution hazard ratio; CI, confidence interval; PY, pack-years.

* Adjusted for age, sex, income, place of residence, hypertension, diabetes mellitus, dyslipidemia, ischemic heart disease, stroke, chronic obstructive pulmonary disease, end-stage kidney disease, and depression. For the health screening subset, body mass index, smoking, alcohol consumption, and regular physical activity were additionally adjusted.

## Discussion

In this large, population-based cohort study, we found that suicide risk among lung cancer patients who underwent surgery was markedly increased during the first postoperative year, with risk subsequently declining and eventually approximating that of matched controls. The temporal patterns differed across treatment modalities and patient subgroups. Patients who received surgery alone experienced a temporary rise in suicide risk that decreased over time, while those treated with adjuvant chemotherapy or radiotherapy exhibited a more sustained elevation in suicide risk, though long-term estimates should be interpreted cautiously given the limited statistical power at later time points.

Our findings build upon previous research indicating that suicide risk among lung cancer patients is highest during the initial postdiagnosis and postoperative period (Rahouma *et al.*, 2018; Zaorsky *et al.*, 2019; Tang *et al.*, 2022; Hofmann *et al.*, 2023). Large population-based analyses of cancer surgery cohorts have also demonstrated that a significant portion of suicides occur within the first year, with cancers characterized by poorer prognoses, such as lung cancer, associated with shorter intervals to suicide. This underscores the early survivorship phase as a period of increased existential distress for these patients (Potter *et al.*, 2023). During this time, patients are frequently confronted with multiple factors that can heighten suicidal vulnerability, including substantial physical symptom burden (pain, dyspnea, and fatigue), treatment-related toxicities, functional deterioration, and financial hardship. These have been linked to increased psychological distress, diminished quality of life, treatment nonadherence, and even reduced survival among individuals with cancer (Boulanger *et al.*, 2022; Deboever *et al.*, 2023; Yu *et al.*, 2023). Each of these factors has been linked to adverse psychological outcomes, including depression and suicidal ideation, in patients with lung cancer.

The notable elevated early risk in patients undergoing adjuvant therapies may be attributed to the combined physical and psychological burdens associated with more intensive treatment regimens. Supporting our observations, previous studies have established chemotherapy and radiotherapy as important predictors of suicide among lung cancer patients (Zhang *et al.*, 2021). These individuals are often diagnosed with advanced-stage disease or possess unfavourable histopathological characteristics, leading to the need for additional postoperative treatments and extended clinical monitoring. It should be noted, however, that TNM staging data were unavailable in the administrative database, and the elevated suicide risk in adjuvant therapy groups may partly reflect the psychological burden of advanced disease and poorer prognosis, rather than, or in addition to, the direct toxic effects of treatment. To partially address this concern, we performed a supplementary analysis stratified by surgical extent as a proxy for disease severity (Supplementary Table S2). Although suicide risk did not differ significantly across resection types (wedge resection, segmentectomy, lobectomy, and pneumonectomy) at any time point, a numerically higher risk was observed with greater surgical extent. This pattern is consistent with a possible contribution of disease severity to suicide risk, though the wide CIs preclude definitive conclusions.

The 5-year recurrence-free survival rates following resection for non-small cell lung cancer (NSCLC) remain limited (87.8% in stage I, 54.7% in stage II, and 33.4% in stage III) (Muraoka *et al.*, 2023), which contributes to persistent prognostic uncertainty. Toxicities associated with adjuvant therapies – including fatigue, neuropathy, anorexia, and specific organ effects – further undermine functional status and overall quality of life. Particularly, the most pronounced early suicide risk appeared in patients treated with postoperative radiotherapy. Acute complications of radiotherapy, such as esophagitis, chest pain, fatigue, and radiation pneumonitis (which affects up to 30% of patients), can significantly interfere with daily activities and increase psychological distress (Farr *et al.*, 2019; Chen *et al.*, 2023). The combined effects of surgery and adjuvant therapies likely contribute to the persistent risk of suicide observed in this patient subgroup.

Another important consideration is the substantial challenges that many patients encounter in adapting to a new normal after undergoing lung cancer treatment. Qualitative studies in resected NSCLC patients indicate that persistent symptoms, fear of recurrence, elevated recovery expectations, and psychosocial stressors – including occupational and financial concerns – frequently hinder reintegration into daily life (Lee *et al.*, 2024). Patients who are inadequately prepared for these changes tend to have greater difficulty coping. Structured discussions regarding anticipated postoperative courses and the timeline for functional recovery have been identified as significant unmet needs (Park *et al.*, 2023; Jung *et al.*, 2024). The broader survivorship literature similarly emphasizes that the posttreatment transition is a unique phase defined by heightened anxiety, disruption of social and occupational roles, uncertainty, and financial toxicity (Institute of Medicine and National Research Council 2006), all of which remain insufficiently addressed in lung cancer follow-up care and could further increase suicidal risk if they are not proactively managed.

Age-stratified analysis demonstrated that younger patients (age 20–49) faced the highest suicide risk at baseline, with this elevated risk tending to persist even 5 years post-surgery. From a clinical perspective, young-onset lung cancer is frequently diagnosed at a later stage and is more often characterized by adenocarcinoma harbouring driver mutations such as epidermal growth factor receptor, which affects the selection of more intensive or prolonged treatment regimens (Skarin *et al.*, 2001; Sumiya *et al.*, 2025). Owing to their generally favourable performance status, these individuals are also more likely to receive aggressive multimodal therapies. These clinical characteristics may contribute to ongoing treatment-related burden and lingering uncertainty regarding prognosis. Concurrently, receiving a cancer diagnosis at a young age can significantly disrupt important life domains such as career development, family planning, and identity formation, and the unexpected onset of disease may exacerbate psychological distress (Laguna *et al.*, 2024). Collectively, these clinical and psychosocial factors may underpin the extended vulnerability to suicide observed in this population subgroup.

Consistent with prior epidemiological studies on suicide (Suominen *et al.*, 2004), male patients exhibited a higher risk of suicide compared with female patients, particularly within the first year after surgery. Males may be less inclined to seek psychological support, which may be attributable to prevailing social expectations concerning masculinity (Hawton, 2000), as well as cultural norms that stress emotional self-control (Payne *et al.*, 2008). Within the Korean social context, where men often identify as primary providers, illness or loss of employment can represent an especially profound emotional adversity. Such circumstances may heighten psychological susceptibility among male patients.

Interestingly, never smokers exhibited an elevated risk of suicide during the first postoperative year. Since lung cancer screening guidelines primarily focus on individuals with a substantial smoking history, never smokers are often diagnosed at a later or more symptomatic stage, which may necessitate more intensive treatment regimens (Khan *et al.*, 2023). Psychologically, unlike smokers who may attribute their diagnosis to prior tobacco use and thus retain some sense of explanatory framework, never-smokers face a diagnosis that is fundamentally inexplicable within their self-narrative. This absence of causal attribution may provoke a more profound existential shock, characterized by disbelief, a sense of injustice, and loss of perceived control, that is qualitatively distinct from the distress experienced by smokers (Chapple *et al.*, 2004). Such responses are consistent with theoretical models of meaning-making in the context of illness, whereby the inability to construct a coherent causal narrative amplifies feelings of helplessness and existential threat (Park, 2010). During the initial treatment phase, when patients simultaneously confront the adverse effects of therapy and profound uncertainty regarding prognosis, this existential burden may be particularly acute, potentially contributing to the elevated suicide risk observed in the first postoperative year among never-smokers.

The present study possesses several important clinical implications. Given that suicide risk is highest during the first postoperative year, screening for distress and suicidality should be embedded at every key clinical transition point, including diagnosis disclosure, preoperative consultation, the initial postoperative follow–up, initiation of chemotherapy/radiotherapy, and first surveillance imaging. Utilizing brief screening instruments with established referral protocols can facilitate a feasible suicide–prevention workflow in oncology settings (Janes, 2018). Early incorporation of supportive care to address physical symptoms, psychological distress, and informational needs is recommended (Kochovska *et al.*, 2020). A targeted and individualized strategy is needed for subgroups at elevated risk, with intervention focus tailored to each group’s specific vulnerabilities. For example, counselling for younger patients should address career disruption and identity loss; for never-smokers, interventions should acknowledge the existential shock arising from the absence of a causal explanation, validating feelings of disbelief and loss of control; and for patients receiving multimodal therapy, the focus should address the cumulative burden of prolonged treatment and the clinical reminder effect of repeated oncologic visits. Providing clear information about conditional survival while implementing a proactive survivorship plan that describes anticipated disease trajectory, follow–up intervals, and typical treatment consequences may help lessen uncertainty and early feelings of hopelessness (Yoo *et al.*, 2021). Incorporating these coordinated psychosocial and informational interventions within lung cancer survivorship programs may aid in mitigating suicide risk in vulnerable patient populations.

This study has certain limitations. First, utilizing administrative data limited access to clinical variables such as stage, histology, or recurrence status. Second, as indicated by prior investigations (Björkenstam *et al.*, 2014), some cases that were recorded as accidental deaths or deaths with undetermined intent may actually represent probable suicides, which could lead to an underestimation of suicide incidence. Third, although pre-existing depression was incorporated as a covariate in the analysis, residual confounding from other unmeasured psychiatric comorbidities such as anxiety disorders or from the absence of social support cannot be fully excluded. Fourth, this cohort was restricted to surgically treated patients, representing a relatively healthier subset of the lung cancer population. Patients with inoperable or advanced-stage disease were not included, and suicide risk in the broader lung cancer population is likely underestimated by the present findings. Lastly, variables such as symptoms of depression or the presence of social support cannot be excluded. Lastly, since this cohort consisted solely of Korean patients, the findings may not be generalizable to other populations (Alothman *et al.*, 2022).

In conclusion, suicide risk among lung cancer patients was highest during the first year following surgery, and the level of risk was influenced by treatment modality and individual patient characteristics. Although the risk gradually decreased in individuals who underwent surgery alone, it persisted at higher levels in those who received adjuvant therapy and in younger patients. Male patients and never smokers also exhibited increased risk in the early postoperative period. These results underscore the need for timely, individualized psychosocial interventions within lung cancer survivorship care, with a particular focus on those at elevated vulnerability.

## Supporting information

10.1017/S2045796026100870.sm001 Yoo et al. supplementary material Yoo et al. supplementary material

## Data Availability

The data that support the findings of this study are available from the Korean National Health Insurance Service, but restrictions apply to the availability of these data, which were used under license for the current study, and so are not publicly available. Data are, however, available at https://nhiss.nhis.or.kr/bd/ay/bdaya001iv.do with the permission of the Korean National Health Insurance Service.

## Supplementary material

The supplementary material for this article can be found at https://doi.org/10.1017/S2045796026100870.

## Table 1 Long description

A baseline characteristics table summarizing the study participants. The mean age of the participants is 64.0 ± 10.0 years, and 65.2% are male. Compared to their matched controls, lung cancer patients are more frequently from the highest income quartile and predominantly live in urban areas. In addition, these patients exhibit a greater prevalence of comorbid conditions, including hypertension, diabetes mellitus, dyslipidemia, ischemic heart disease, stroke, chronic obstructive pulmonary disease, and depression.

Navigate back to Table 1.

## Table 2 Long description

A table presenting subdistribution hazard ratios (sHR) for suicide risk across different post-surgery periods. In the total population, lung cancer patients have a significantly higher risk of suicide during the first year after surgery (sHR 1.88) compared to matched controls, which peaks remarkably in the surgery plus radiotherapy group (sHR 7.00). However, after 1 year, the overall suicide risk among lung cancer patients decreases below the control level (sHR 0.78 after 1 year, 0.75 after 3 years, and 0.72 after 5 years), driven primarily by the surgery-only group. Similar trends of an initial high risk in the first year followed by a subsequent decline are consistently observed in the health screening subset.

Navigate back to Table 2.

## Table 3 Long description

A table analysing suicide risk stratified by subgroups across different post-surgery periods. In the age-stratified analysis, patients aged 20–49 years demonstrate the highest baseline suicide risk (sHR 2.02, *P* for interaction 0.018), which remains relatively increased throughout the follow-up period (sHR 1.90 after 1 year, sHR 1.61 after 5 years). Both male and female patients show an increased suicide risk in the initial year following surgery, with the association being more pronounced among males (sHR 1.93). Within the health screening subset, never-smokers exhibit a higher suicide risk during the first postoperative year (sHR 2.21), with a generally decreasing trend observed across all smoking status groups as time progresses.

Navigate back to Table 3.
